# Analysis of genome-wide knockout mouse database identifies candidate ciliopathy genes

**DOI:** 10.1038/s41598-022-19710-7

**Published:** 2022-12-01

**Authors:** Kendall Higgins, Bret A. Moore, Zorana Berberovic, Hibret A. Adissu, Mohammad Eskandarian, Ann M. Flenniken, Andy Shao, Denise M. Imai, Dave Clary, Louise Lanoue, Susan Newbigging, Lauryl M. J. Nutter, David J. Adams, Fatima Bosch, Robert E. Braun, Steve D. M. Brown, Mary E. Dickinson, Michael Dobbie, Paul Flicek, Xiang Gao, Sanjeev Galande, Anne Grobler, Jason D. Heaney, Yann Herault, Martin Hrabe de Angelis, Hsian-Jean Genie Chin, Fabio Mammano, Chuan Qin, Toshihiko Shiroishi, Radislav Sedlacek, J.-K. Seong, Ying Xu, The IMPC Consortium, Arthur L. Beaudet, Bob Braun, Natasha Karp, Ann-Marie Mallon, Terrence Meehan, Yuichi Obata, Helen Parkinson, Damian Smedley, Glauco Tocchini-Valentini, Sara Wells, K. C. Kent Lloyd, Colin McKerlie, Ala Moshiri

**Affiliations:** 1 The University of Miami Leonard M. Miller School of Medicine, Miami, FL 33136 USA; 2 Department of Small Animal Clinical Sciences, University of Florida, College of Veterinary Medicine, Gainesville, FL 32608 USA; 3 The Centre for Phenogenomics, Toronto, ON Canada; 4 Lunenfeld-Tanenbaum Research Institute, Mount Sinai Hospital, Toronto, ON M5G 1X5 Canada; 5 Covance Inc, Chantilly, VA 20151 USA; 6 Mouse Biology Program, U.C. Davis, Davis, CA 95618 USA; 7 The Hospital for Sick Children, 555 University Avenue, Toronto, ON M5G 1X8 Canada; 8 The Wellcome Trust Sanger Institute, Wellcome Genome Campus, Hinxton, Cambridge, CB10 1SA UK; 9 Centre of Animal Biotechnology and Gene Therapy (CBATEG), Universitat Autònoma de Barcelona, 08193 Barcelona, Spain; 10 The Jackson Laboratory, Bar Harbor, ME 04609 USA; 11 Medical Research Council Harwell Institute (Mammalian Genetics Unit and Mary Lyon Centre), Harwell Campus, Oxfordshire, OX11 0RD UK; 12 Department of Molecular and Human Genetics, Baylor College of Medicine, Houston, TX 77030 USA; 13 Phenomics Australia, The Australian National University, 131 Garran Rd, Acton, Canberra, ACT 2601 Australia; 14 European Molecular Biology Laboratory, European Bioinformatics Institute, Wellcome Genome Campus, Hinxton, Cambridge, CB10 1SD UK; 15 SKL of Pharmaceutical Biotechnology and Model Animal Research Center, Collaborative Innovation Center for Genetics and Development, Nanjing Biomedical Research Institute, Nanjing University, Nanjing, 210061 China; 16 Indian Institutes of Science Education and Research, Dr. Homi Bhabha Rd, Ward No. 8, NCL Colony, Pashan, Pune, Maharashtra 411008 India; 17 Faculty of Health Sciences, PCDDP North-West University, North-West University Potchefstroom Campus 11 Hoffman Street, Potchefstroom, 2531 South Africa; 18 Institut de Génétique et de Biologie Moléculaire et Cellulaire, Université de Strasbourg, 67400 Illkirch, France; 19 German Mouse Clinic, Institute of Experimental Genetics, Helmholtz Zentrum München, German Research Center for Environmental Health, Ingolstädter Landstraße 1, 85764 Neuherberg, Germany; 20 National Laboratory Animal Center, National Applied Research Laboratories (NARLabs), 3F., No. 106, Sec. 2, Heping E. Rd., Da’an Dist., Taipei City, 106214 Taiwan (R.O.C.); 21 Monterotondo Mouse Clinic, Italian National Research Council (CNR), Institute of Cell Biology and Neurobiology, Adriano Buzzati-Traverso Campus, Via Ramarini, 00015 Monterotondo Scalo, Italy; 22 National Laboratory Animal Center, National Applied Research Laboratories (NARLabs), Beijing, China; 23 RIKEN BioResource Center, Tsukuba, Ibaraki 305-0074 Japan; 24 Czech Center for Phenogenomics, Institute of Molecular Genetics of the Czech Academy of Sciences, IMG BIOCEV Building SO.02 Prumyslova 595, 252 50 Vestec, Czech Republic; 25 Korea Mouse Phenotyping Consortium (KMPC) and BK21 Program for Veterinary Science, Research Institute for Veterinary Science, College of Veterinary Medicine, Seoul National University, 599 Gwanangno, Gwanak-gu, Seoul, 08826 South Korea; 26 CAM-SU Genomic Resource Center, Soochow University, Organization Planning of No. 1 Shizi Street, Suzhou, 215123 China; 27 Department of Surgery, School of Medicine, U.C. Davis, Sacramento, CA 95817 USA; 28 Department of Laboratory Medicine and Pathobiology, Hospital for Sick Children (SickKids), The Centre for Phenogenomics, Faculty of Medicine, University of Toronto, 25 Orde Street, Toronto, ON M5T 3H7 USA; 29 Department of Ophthalmology and Vision Science, School of Medicine, U.C. Davis Eye Center, 4860 Y. Street, Suite 2400, Sacramento, CA 95817 USA; 30 University of Reno, Nevada, School of Medicine, Reno, NV 89557 USA; 31 Comparative Pathology Laboratory, U.C. Davis, Davis, 95616 USA; 32 Institut de Génétique et de Biologie Moléculaire et Cellulaire, Université de Strasbourg, 1 rue Laurent Fries, 67404 Illkirch, France; 33 Centre National de la Recherche Scientifique, UMR7104, Illkirch, France; 34 Institut National de la Santé et de la Recherche Médicale, U1258, Illkirch, France; 35 Université de Strasbourg, 1 rue Laurent Fries, 67404 Illkirch, France; 36 CELPHEDIA, PHENOMIN, Institut Clinique de la Souris (ICS), CNRS, INSERM, Université of Strasbourg, 1 rue Laurent Fries, 67404 Illkirch-Graffenstaden, France; 37 Clinical Pharmacology, Charterhouse Square, Barts and the London School of Medicine and Dentistry, Queen Mary University of London, London, EC1M 6BQ UK; 38 Institute of Laboratory Animal Sciences, Chinese Academy of Medical Science, 5 Panjiayuan Nanli, Chaoyang District, Beijing, 100021 China

**Keywords:** Cell biology, Organelles, Molecular medicine, Ciliogenesis

## Abstract

We searched a database of single-gene knockout (KO) mice produced by the International Mouse Phenotyping Consortium (IMPC) to identify candidate ciliopathy genes. We first screened for phenotypes in mouse lines with both ocular and renal or reproductive trait abnormalities. The STRING protein interaction tool was used to identify interactions between known cilia gene products and those encoded by the genes in individual knockout mouse strains in order to generate a list of “candidate ciliopathy genes.” From this list, 32 genes encoded proteins predicted to interact with known ciliopathy proteins. Of these, 25 had no previously described roles in ciliary pathobiology. Histological and morphological evidence of phenotypes found in ciliopathies in knockout mouse lines are presented as examples (genes *Abi2, Wdr62, Ap4e1, Dync1li1,* and *Prkab1*). Phenotyping data and descriptions generated on IMPC mouse line are useful for mechanistic studies, target discovery, rare disease diagnosis, and preclinical therapeutic development trials. Here we demonstrate the effective use of the IMPC phenotype data to uncover genes with no previous role in ciliary biology, which may be clinically relevant for identification of novel disease genes implicated in ciliopathies.

## Introduction

The pathophysiology of ciliopathies reflects abnormalities in the function of both primary and motile cilia. Primary cilia dyskinesia (PCD) is a multi-syndromic disorder caused by defects in motile cilia, including chronic otosinopulmonary disease, *situs* abnormalities, and male infertility. PCD results from an inability of motile cilia to transport mucus through the respiratory tract and nasal sinuses resulting in persistent infection^1^. *Situs* abnormalities are characterized by lack of normal left–right axis asymmetry. Within the embryonic node there are monociliated cells that beat, moving extracellular fluid and morphogens in order to establish normal left–right axis asymmetry. These developmental abnormalities are also a result of altered cilia motility^2^. Male infertility is caused by an abnormal flagellum, itself a modified motile cilia, that affects the progressive and non-progressive motility of sperm^3,4^. Male infertility can also be caused by loss of motile ciliary action within the efferent ductules of the testes and/or the ciliated epithelial lining of efferent epididymal ductules that are responsible for moving spermatozoa to the epididymis for final maturation^5^.

Two hallmark features of abnormal structure and function of primary cilia include aberrant kidney function and retinal degeneration^6^. Each renal tubular epithelial cell contains a single primary cilium that can act in mechano- and chemo-sensation. Polycystic kidney disease (PKD) and nephronophthisis (NPHP) are diseases in which normal renal architecture is replaced by cysts caused by abnormalities in the primary cilium of renal epithelial cells^7^. Mutations of proteins that are components of the primary cilium, for example PKD1, PKD2, and PKHD1, are associated with PKD, while mutations of the ciliary proteins NPHP1 and INVS are associated with NPHP^6^.

Ciliopathies that commonly have symptoms of retinal degeneration include Leber Congenital Amaurosis (LCA), NPHP, Senior-Loken Syndrome (SLS), Joubert Syndrome (JBTS), and Bardet–Biedl Syndrome (BBS)^8^. While single gene mutations exist that are unique to each syndrome, several syndromes can share a single genetic basis. For example, CEP290 has been linked to NPHP, BBS, SLS, and JBTS^8–12^. CEP290 is a known centrosomal protein that is found in renal epithelial cells and retinal photoreceptors^13^. Outside of retinal and renal phenotypes, skeletal anomalies such as polydactyly can also develop as is the case with the ciliopathy Meckel–Gruber syndrome (MGS)^14^. The clinical spectrum of ciliopathies can include hydrocephalus, intellectual disability, craniofacial defects, cardiac anomalies, lung disease, skeletal abnormalities, liver and pancreatic cysts, kidney disease, and reduced or total infertility.

Considerable effort has been directed toward understanding the scope of proteins interacting with cilia, both directly and through secondary protein interactions, in order to more fully understand potential disease causing mutations that encompass ciliopathies^15–17^. These studies have shed light on new disease-causing or disease-contributing genes associated with ciliopathies^15–17^. However, in the discovery of such genes it is useful to validate predicted ciliopathy proteins or interacting proteins in an in vivo model system. Knockout mice that express a null “loss of function” allele for a gene can determine if mutations in predicted genes lead to the expected phenotype and are useful diagnostically in clinical cases of human ciliopathies, including forms of pediatric ophthalmic pathology.

The International Mouse Phenotyping Consortium (IMPC) is a mouse and phenotype data resource for single gene knockout (KO) mouse lines. Since its inception it has expanded to 20 research centers with expertise in high throughput KO mouse production and phenotyping^18^. To date, production and comprehensive phenotyping has been completed for 6,440 unique genes in IMPC data release 11.0 queried 29 April 2020. Cohorts of KO mice (at least 7 female and 7 male mice per gene) are analyzed and studied using a sequential set of phenotyping tests starting at 4 weeks of age and a set of terminal tests at 16 weeks of age that includes necropsy, tissue collection, and histology using standardized protocols^19,20^. Standardization and harmonization of testing protocols and data analysis are performed by the IMPC Data Coordination Center (DCC) to identify and confirm phenodeviants^21,22^. Given the characteristic presentation of ciliopathies with functional or morphological abnormalities in eyes, kidneys, and reproductive tract, we queried the IMPC database for all mouse lines with abnormal ocular traits and at least one other ciliopathy-associated system: renal or reproductive.

We present novel genes in KO mouse lines that exhibit phenotypes consistent with ciliopathies. Further, we present evidence indicating how loss-of-function mutations in these genes are related to the dysfunction of primary or motile cilia. Candidate ciliopathy genes presented here are of potential interest for further investigation of their association to the cilium and to confirm their association with clinical ciliopathies, particularly those responsible for pediatric eye disease.

## Results

### Query of the IMPC database for abnormal traits associated with ciliopathies

**Figure 1:**
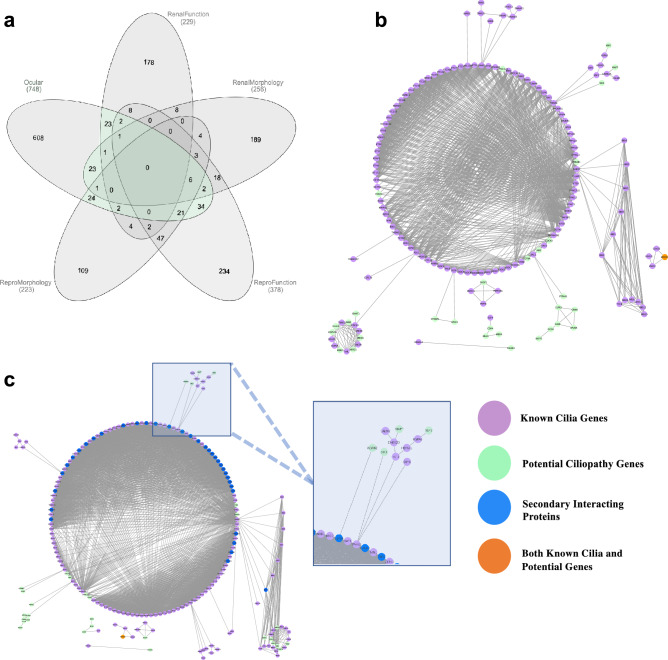
Identification of potential ciliopathy genes and protein network interactions of potential ciliopathy genes and known ciliopathy proteins. (**a**) Venn diagram showing intersection of IMPC genes associated with various phenotype subgroups, those highlighted in green outline have ocular and one or more other concomitant renal or reproductive trait abnormality and are subsequently considered “Potential ciliopathy genes” (**b**) STRING interaction map of known ciliopathy genes with potential ciliopathy genes. Proteins are shown as nodes on the map where green is indicative of a potential ciliopathy gene, purple shown known or gold standard ciliopathy genes, orange are those that were identified to be potential ciliopathy genes and are previously known as ciliopathy genes. Each line represents a STRING interaction score of greater than or equal to .9 based on 5 pieces of evidence: “experiments,” “databases,” “co-expression,” “neighborhood,” “gene fusion,” and “co-occurrence.” 24 of the 140 potential ciliopathy genes are shown to be directly interacting with known ciliopathy genes. (**c**) Incorporation of additional proteins or nodes into the network where blue nodes are genes not found in either gold standard or potential ciliopathy genes. Highlighted is an interacting oculorenal gene *WDR62* which is predicted to interact with known ciliopathy genes through an additional protein CEP63.

The IMPC database contained KOs for 748 genes which exhibited abnormal ocular phenotype(s) identified by eye examination, optic imaging, and/or histopathology (“ocular genes”). Of these, 256 demonstrated abnormal renal morphology at necropsy (“kidney morphology genes”). 132 had abnormal values for blood urea nitrogen (BUN) and 110 had abnormal plasma creatinine (CRE); collectively (“renal function genes”). Of the total 229 renal function genes, 13 were found to have altered levels of both BUN and CRE. Abnormal reproductive tract morphology was identified at necropsy and/or histopathology (“reproductive morphology genes”) in 223 lines. Abnormal reproductive phenotypes was identified by fertility analysis (“reproductive function genes”) in 378 lines. These groups are summarized in Fig. 1a.

### Identification of potential ciliopathy genes

Query of the IMPC database for five different categories of traits associated with ciliopathies led to five distinct gene sets: “ocular genes”, “kidney morphology genes”, “kidney function genes”, “reproductive tract morphology genes”, and “reproductive tract function genes”. To identify “potential ciliopathy genes” with a particular focus on those associated with congenital blindness syndromes, all “kidney morphology genes”, “kidney function genes”, “reproductive tract morphology genes”, and “reproductive tract function genes” were then assessed for concomitant ocular trait abnormalities. If a line had one ocular and any renal or reproductive system abnormality then it was considered to be a “potential” ciliopathy gene. A total of 140 unique potential ciliopathy genes were identified (Fig. 1a, listed in Supplementary Table S2). Phenotypes associated with each gene can be found in Table S2. Of these 140 lines, clinically relevant morphological or functional traits in all three systems (ocular, renal and reproductive) included a total of 31 genes: *Abce1, Abhd11, Abi2, Ap4e1, Aplnr, Atp8b1, Bambi, Chsy3, Cops7a, Dnase1l2, Edc3, Fndc3b, Folr1, Gcgr, Gkn1, Gpa33, Ica1, Mink1, Mmachc, Nacc1, Pgbd1, Ptp4a1, Pygo2, Rspo1, Rxfp2, Sik3, Spred3, Sra1, Sun1, Tmem160, Tmem30b,* and *Wdr62.* Detailed phenotype abnormalities can be found in Table S2.

### Comparison of potential ciliopathy genes with preexisting cilia gene databases

From the IMPC 11.0 release, queried 29 April 2020, 5284 mouse lines had completed all phenotyping and were analyzed. Of these, 5190 lines had a corresponding human ortholog: 235 IMPC 11.0 genes were found in the 830 CiliaCarta genes^17^. 85 of the 217 Syscilia database genes were found in the IMPC 11.0 release. The 235 CiliaCarta genes and the 85 Syscilia genes that have complete data in the IMPC 11.0 release were analyzed for presence of phenotypic abnormalities consistent with ciliopathies. This data is summarized in Table S3. Of our 140 potential ciliopathy genes only 2 were found in the CiliaCarta database, *Mapt* and *Myo7a*. One of the potential ciliopathy genes from our analysis was also found in the list of known cilia genes based on the Syscilia database, *Myo7a*. (Fig. 1b,c).

### Identification of candidate ciliopathy genes by protein–protein interaction analysis

We sought to determine if any of the 140 potential ciliopathy genes protein product interacted with known ciliopathy proteins. For the gold standard or known ciliopathy protein list 217 genes identified by Boldt et al.^15^ as part of the Syscilia were used. Figure 1b shows that of the 140 potential ciliopathy genes protein products, 24 are predicted to interact directly with known cilia proteins. Mouse genes were transposed to their human orthologs for the analysis. The human orthologs of the 24 corresponding mouse genes, *Abi2, Ap4e1, Cdc42, Cdk4, Celsr1, Cops7a, Cyba, Dtnbp1, Dync1li1, Ehmt1, Folr1, Herc1, Klhl5, Mapt, Mib2, Myo7a, Prkab1, Ptp4a1, S1pr3, Sik3, Sugp1, Ube2a, Wsb2, and Xbp1,* were each predicted to interact directly known cilia proteins. To further test if any of the potential ciliopathy genes had known interactions with ciliopathy proteins, 35 interacting proteins predicted by StringDB but not part of either of our 140 potential ciliopathy genes list nor the known 217 cilia gene list were incorporated into the interaction network. This analysis revealed one additional oculorenal gene predicted to interact with the cilium through secondary protein interactions, *Wdr62,* shown in Fig. 1c. In addition, 7 genes had primary interactions with the 24 directly interacting potential ciliopathy genes and thus had secondary interactions with known cilia proteins: *Aplnr, Casr, Gcgr, Grm6, Med1(or Mbd4), Mlh1,* and *Rxfp2* (Fig. 1b). In total the 32 genes with evidence to have either primary or secondary interactions with known ciliopathy proteins and were considered to be “candidate ciliopathy genes” summarized in Table S1.

### Candidate ciliopathy genes and literature review

The 32 candidate ciliopathy genes were reviewed for a previously published role in cilia biology in vertebrates or known association with ciliopathy in humans. Seven of the 32 have been previously identified as ciliopathy genes in vertebrates. However, only one gene of the 32 has been shown to have function in human cilia, leaving 31 candidate human ciliopathy genes and 25 genes with a previously undescribed role in ciliary biology. The summary of the literature review can be found in Table S1.

### Histologic evidence of knockout mouse strains with concomitant abnormal ocular and renal or reproductive phenotypes

**Figure 2:**
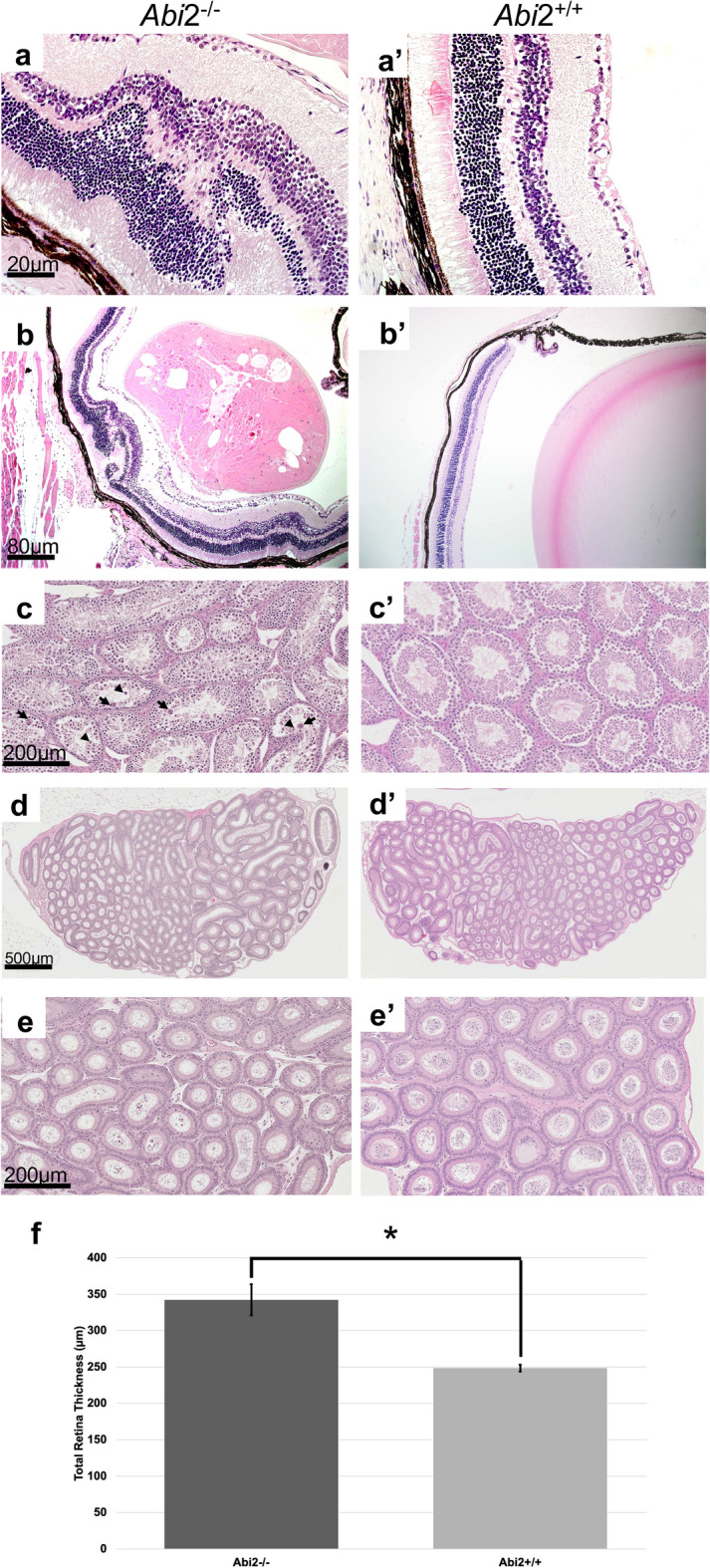
*Abi2*^−/−^ mice had eye and male reproductive tract abnormalities consistent with ciliopathy compared to controls. (**a**) Marked, chronic, multifocal localized retinal dysplasia characterized by multiple clusters of external nuclear structures within the outer plexiform layer. (**b**) Cortical cataract is present with marked, chronic, focally extensive swollen and disrupted lens fibers with abnormally retained nuclei (balloon cells) with capsular thickening and wrinkling in the cortical region of the lens. (**c**) 20 × magnification histopathology showed testicular degeneration, marked, chronic, bilateral, multifocal vacuolation of the seminiferous tubule epithelia with primary and secondary spermatocyte and spermatid hypocellularity, with very few spermatozoa. Apoptotic bodies (arrowheads) and multinucleated giant cells (arrows) were frequent. (**d**) On magnification (5 ×), the epididymis showed marked hypospermia. Epididymal ducts in all segments of the epididymis (caput, corpus, cauda) contained cell and protein debris in the lumen with few mature spermatozoa. (**e**) Magnification (20 ×) of (**d**). (**f**) Quantitative measurement of average total retina thickness, where *Abi2*^+*/*+^ n = 12, average = 342.35 µm, SE = 4.88 and *Abi2*^−/−^ n = 9, average = 342.35 µm, SE = 21.35 *p*-value = 0.0190. All error bars represent standard error of the mean, * indicates *p*-value < .05 result of student’s two-tailed t-test.

Five examples of the 32 candidate ciliopathy genes are presented. Mice with a homozygous KO of *Abi2* show both eye and male reproductive abnormalities consistent with ciliopathies. The ophthalmic abnormalities seen in *Abi2* null mice involve both the retina and the lens. Specifically, marked, chronic, multifocal localized retinal dysplasia characterized by multiple clusters of external nuclear structures are present within the outer plexiform layer of the retina. The lens features include cortical cataract with marked, chronic, focally extensive swollen and disrupted lens fibers with abnormally retained nuclei (balloon cells), as well as capsular thickening and wrinkling in the cortical region of the lens (Fig. 2a,b). Necropsy identified small testis, which on histopathology were characterized by testicular degeneration, marked, chronic, bilateral, multifocal vacuolation of the seminiferous tubules with hypocellularity, sparse spermatids, and very few spermatozoa. (Fig. 2c,d). The epididymis showed marked chronic hypospermia. All segments of epididymal ducts contain round bodies in the lumen with few spermatozoa (Fig. 2e). Quantitative analysis of the retinas of *Abi2*^−/−^ mice revealed a total increased retinal thickness consistent with the noticeable dysplasia. (Fig. 2f).

**Figure 3:**
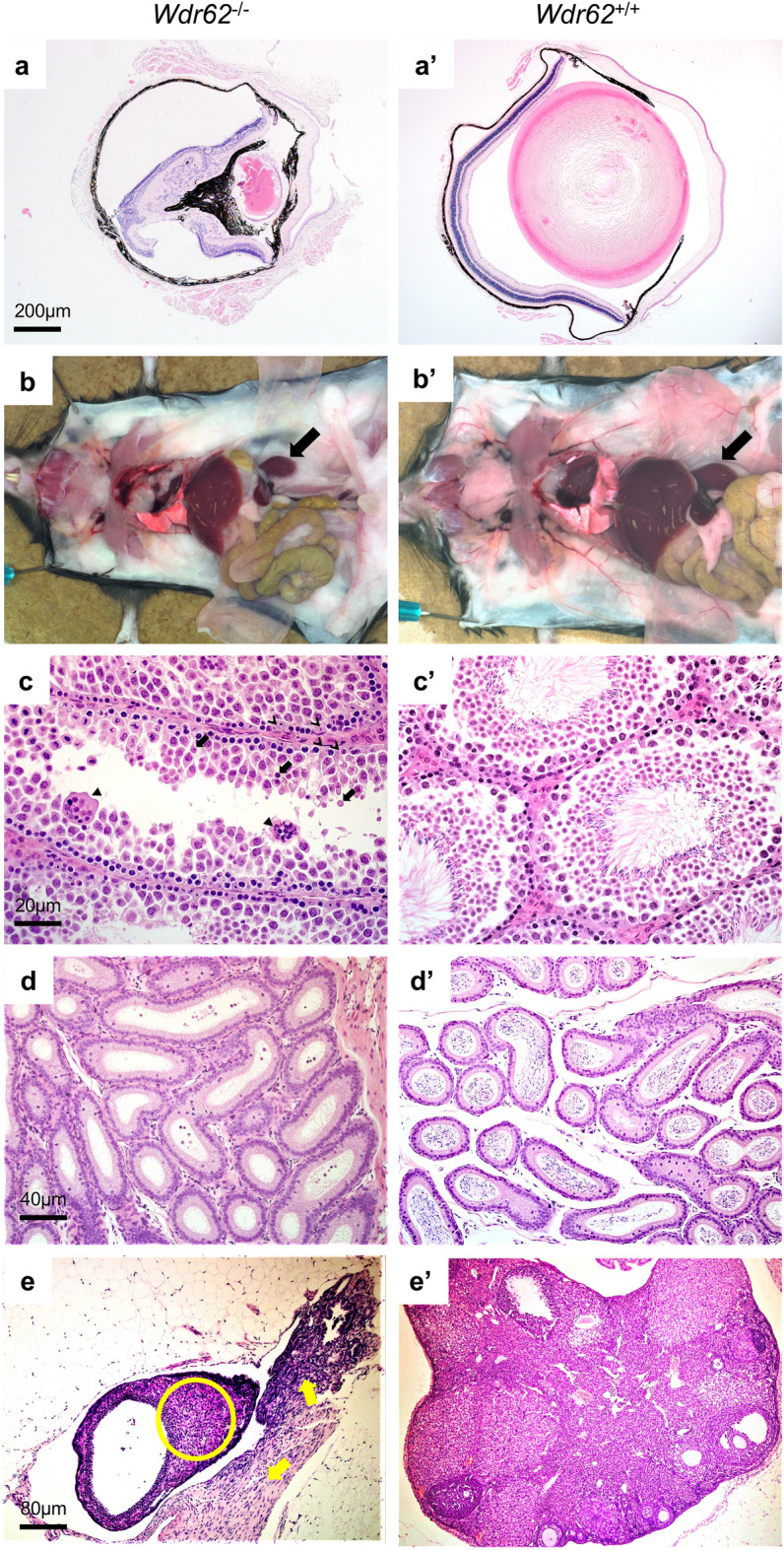
*Wdr62*^−/−^ mice have ocular, renal, and reproductive organ abnormalities consistent with ciliopathy. (**a**) Microphthalmia with undeveloped lens and residual lens vesicle remnant in the vitreous cavity. The ocular contents are small, malformed, and disorganized. (**b**) Small kidneys at necropsy (arrow), but histologically normal in appearance (data not shown). (**c**) Testis with seminiferous tubule degeneration characterized by defective spermatogenesis and aspermia with increased apoptosis of meiosis I spermatocytes (arrow), and multinucleated syncytia (solid arrowhead). There are numerous spermatogonia and spermatocytes, but rare spermatids and mature spermatozoa. Sertoli cells are prominent (line arrowhead) and seminiferous tubules contain degenerated cells with irregular dense nuclei. (**d**) Epididymal ducts containing scattered cell debris and no spermatozoa. (**e**) Ovaries are small, hypoplastic with no detectable folliculogenesis. The ovarian bursa is hyperplastic, dominated by fibrous connective tissue stroma with spindle shaped cells (solid yellow arrows) and rare immature solid tubular structures (yellow circles) representing mesonephric remnants in the ovary. Germinal epithelium, oocytes, and follicles are absent.

*Wdr62* homozygous KO mice had ocular, renal, and reproductive organ abnormalities consistent with ciliopathy. The eyes demonstrated microphthalmia with undeveloped lens and residual lens vesicle remnant in the vitreous cavity. The ocular contents were small, malformed, and disorganized (Fig. 3a). The kidneys were small at necropsy, but constitutively unremarkable histologically (Fig. 3b). In the testes there was defective spermatogenesis with aspermia. There were numerous spermatogonia and spermatocytes, but rare spermatids and mature spermatozoa in seminiferous tubules of testes. Sertoli cells were prominent in the interstitium and seminiferous tubules contained degenerated cells with irregular dense nuclei. Overall, there was decreased testis size, reductions in spermatocyte and spermatid number, increased apoptosis of meiosis I spermatocytes, and multinucleated syncytia (Fig. 3c). Epididymal ducts also show defects with scattered cell debris and no spermatozoa (Fig. 3d). Homozygous deletion of *Wdr62* also had effects on female reproductive organs. The ovaries were hypoplastic with no apparent folliculogenesis. The ovaries and ovarian bursa were very small dominated by fibrous connective tissue stroma with spindle shaped cells. Germinal epithelium, oocytes, and follicles were absent. The fibrous stroma contained immature solid tubular structures representing mesonephric remnants and interstitial hyperplasia (Fig. 3e).

**Figure 4:**
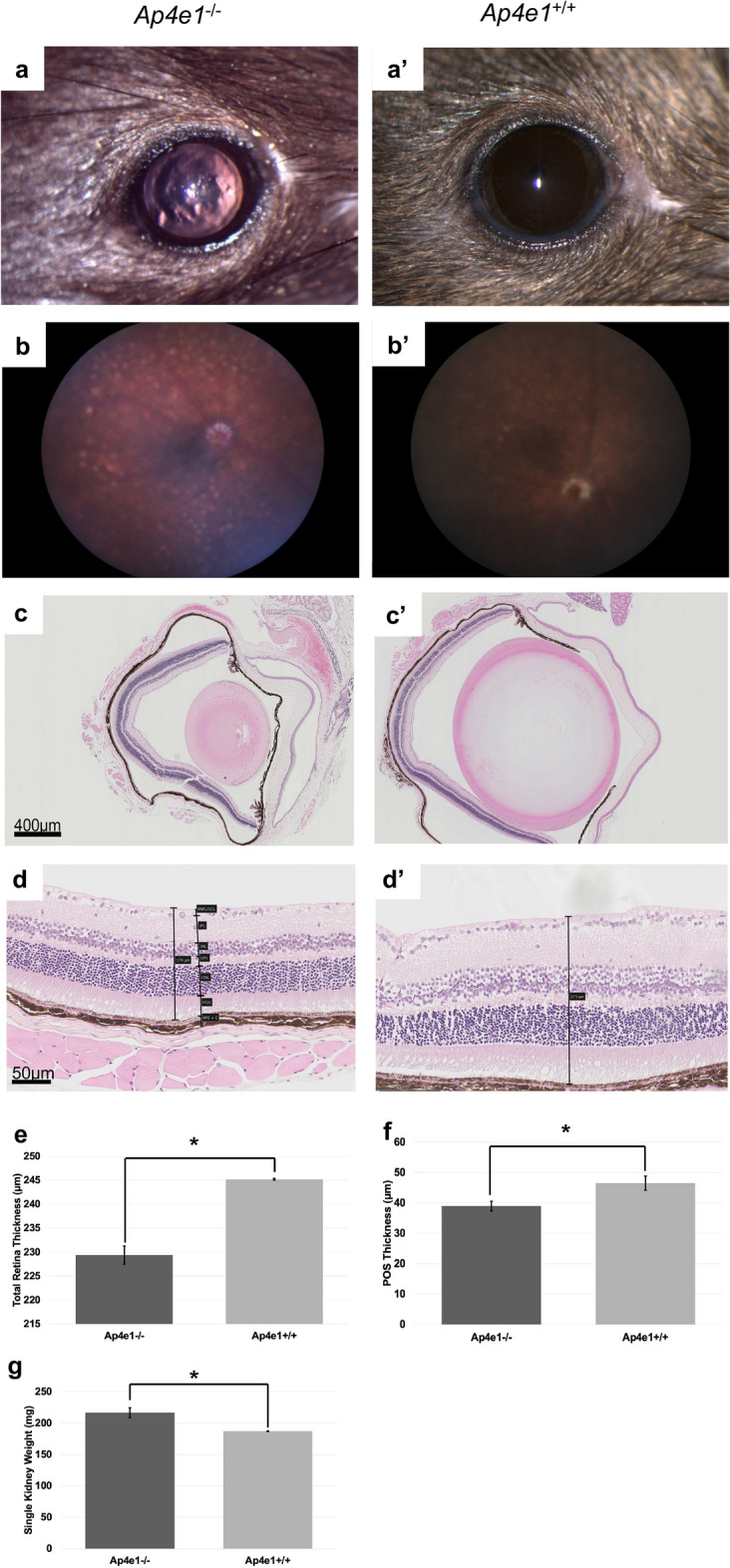
*Ap4e1*^−/−^ mice have ocular abnormalities in the retina, cornea, lens, and have small kidneys. (**a**) Corneal epithelial irregularity on gross examination of the eye, focally extensive corneal epithelial squamous hyperplasia. (**b**) Extensive retinal hypopigmentary lesions on color fundus photography. (**c**) Abnormal lens morphology. (**d**) Decreased total retinal thickness. Retina layers labeled where RNFL/GCL, retinal nerve fiber layer/ganglion cell layer; IPL, inner plexiform layer; INL, inner nuclear layer; OPL, outer plexiform layer; ONL, outer nuclear layer; POS, photoreceptor outer segments; RPE, retinal pigmented epithelium; C, choroid. (**e**) Quantitative measurement of average total retina thickness, where *Ap4e1*^+*/*+^ n = 3861, average = 245.20 µm, SE = 0.164 and *Ap4e1*^−/−^ n = 45, average = 229.39 µm, SE = 1.89, *p*-value = 7.78 × 10^–11^. (**f**) Quantitative measurement of POS layer thickness. Where *Ap4e1*^+*/*+^ n = 8, average = 46.54 µm, SE = 2.35 and *Ap4e1*^−/−^ n = 16, average = 38.99 µm, SE = 1.58, *p*-value = 0.019 . (**g**) Quantitative measurement of single kidney weight where *Ap4e1*^+*/*+^ n = 3629, 187.18 mg, SE = 0.581 and *Ap4e1*^−/−^ n = 29, 217.54 mg, SE = 7.85, *p*-value = 0.00085. All error bars represent standard error of the mean, * indicates *p*-value < .05 result of student's two-tailed t-test.

**Figure 5:**
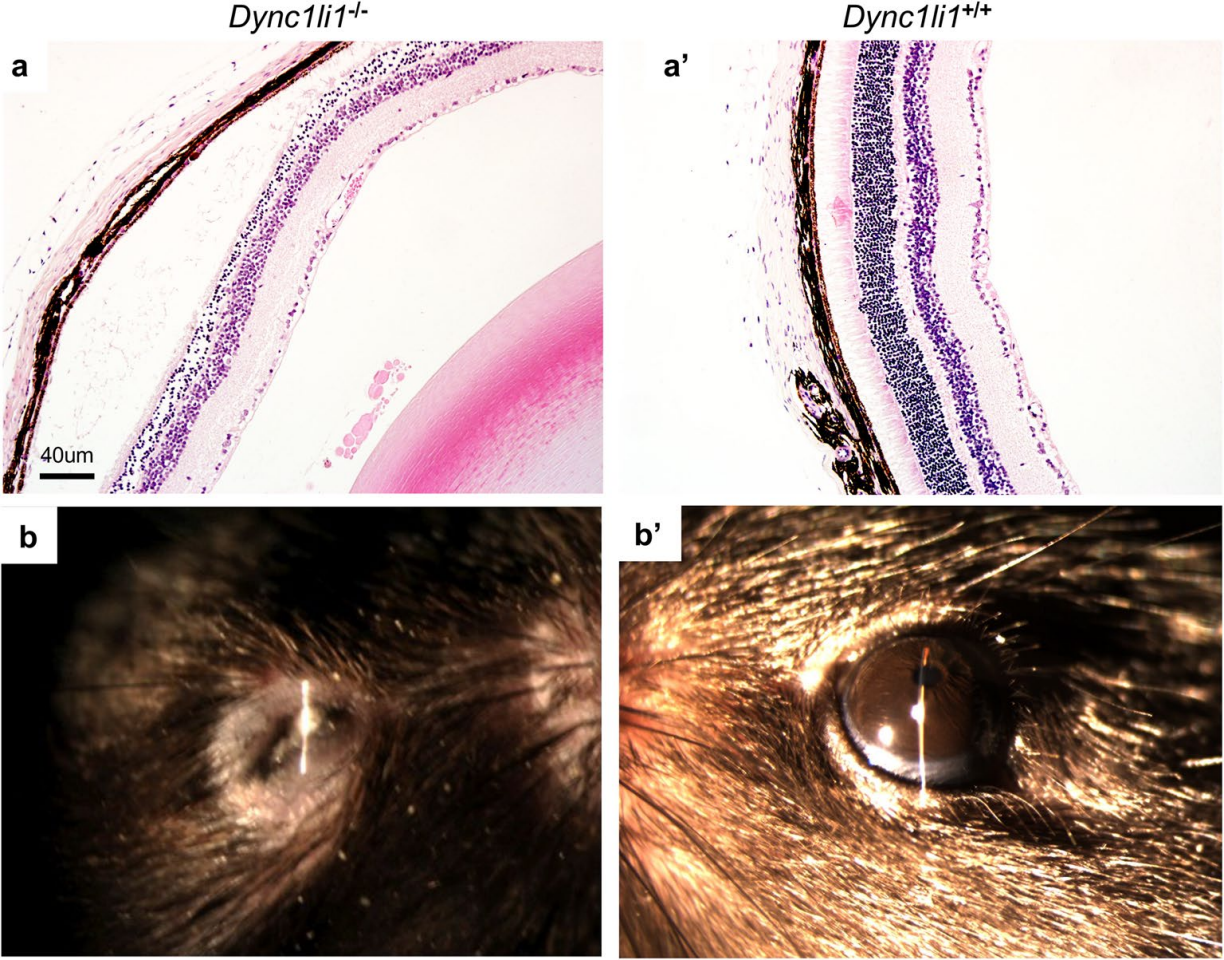
*Dync1li1*^−/−^ mice have ocular abnormalities in the retina and microphthalmia. (**a**) Extensive retinal hypoplasia, atrophy, and cellular disorganization across all layers of the retina. In addition, the retina has decreased total thickness compared to control. (**b**) Microphthalmia evident on slit lap examination compared to control.

**Figure 6:**
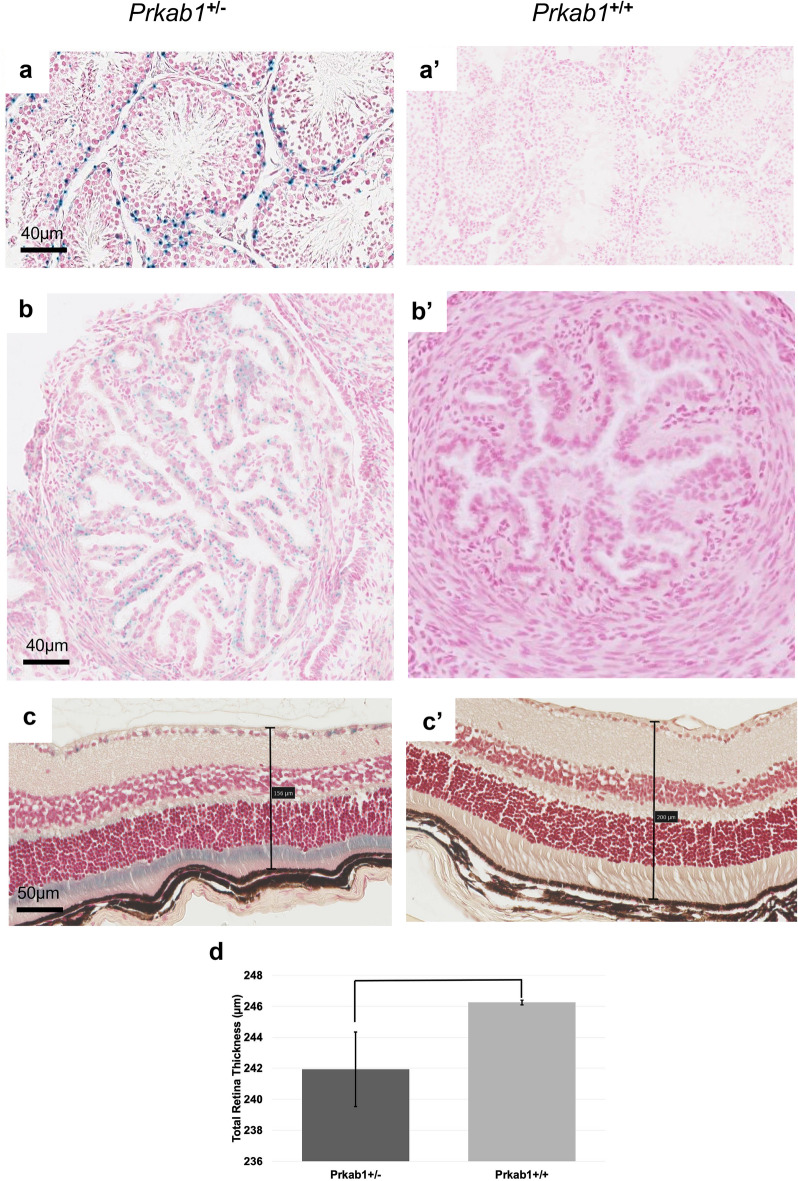
*Prkab1*^+/−^ LacZ expression under *Prkab1* promoter is found in multiple reproductive tissues and photoreceptor outer segments of the retina. (**a**) lacZ reporter shows expression in the spermatogonia layer of the testis. (**b**) lacZ reporter expression under the control of the *Prkab1* promoter in the oviduct shows faint scattered expression. (**c**) lacZ reporter expression under the control of the *Prkab1* promoter shows strong signal in the wavy and disorganized photoreceptor inner and outer segments. (**d**) Quantitative measurement of average total retina thickness, where *Prkab1*^+*/*+^ n = 3698, average = 246.25 µm, SE = .148 and *Prkab1*^+/-^ n = 28, average = 241.93, SE 2.40, *p*-value = 0.084. No significant difference was found in total retinal thickness based on student’s two-tailed t-test. All error bars represent standard error of the mean.

**Figure 7:**
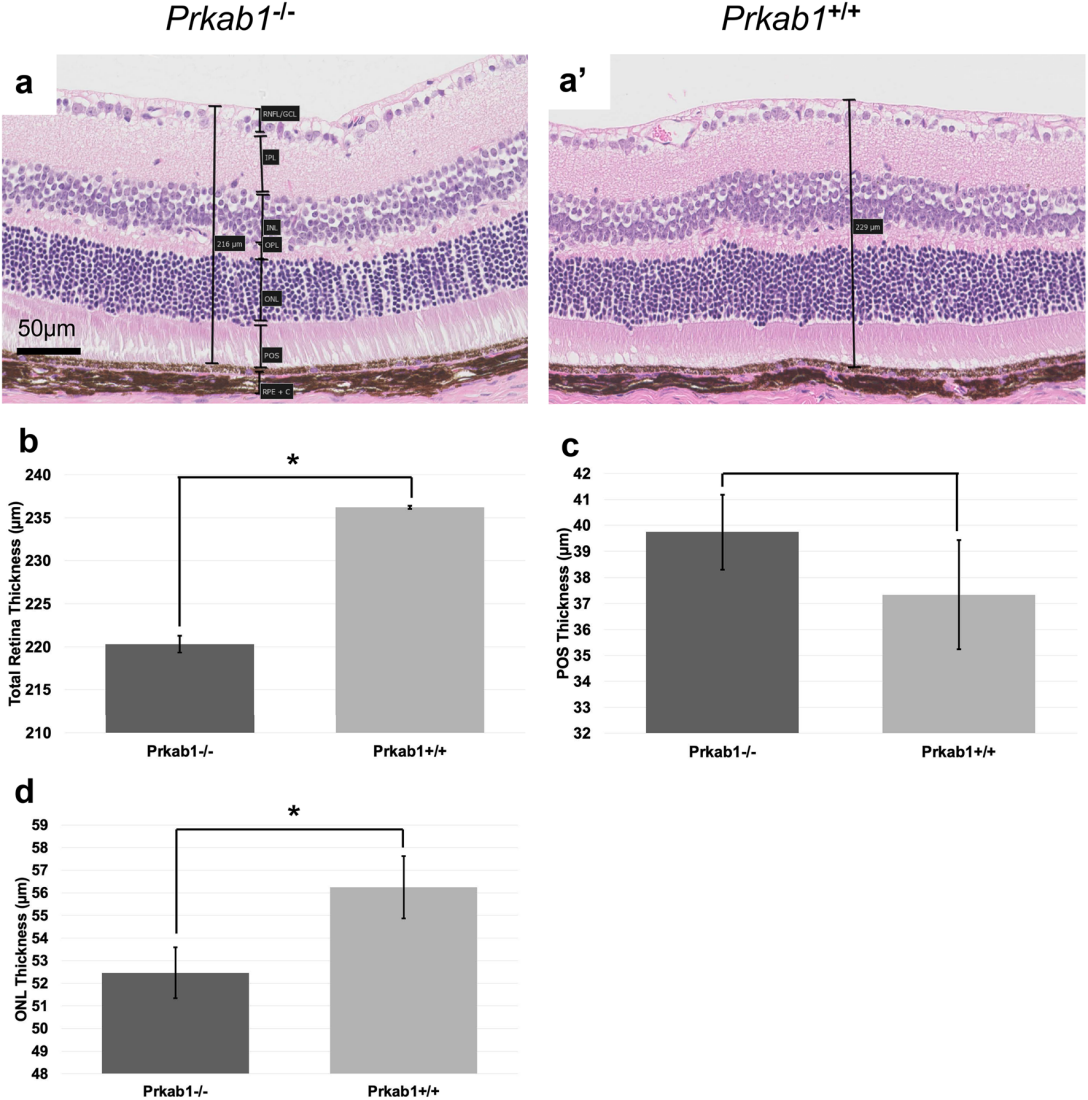
*Prkab1*^−/−^ mice have decreased total retinal thickness and reduced thickness of the ONL. (**a**) *Prkab1*^−/−^ mice retinas show decreased total thickness with evidence of reduced thickness in the ONL. (**b**) Quantitative measurement of average total retina thickness, where *Prkab1*^+*/*+^ n = 6567, average = 236.21 µm, SE = 0.19 and *Prkab1*^−/−^ n = 85, average = 220.29 µm, SE = .998. *p* = 1.078 × 10^–27^ (**c**) Quantitative measurement of POS layer thickness where *Prkab1*^+*/*+^ n = 9, average = 37.33 µm, SE = 2.09 and *Prkab1*^−/−^ n = 14, average = 39.74 µm, SE = 1.43 *p*-value = .357 (**d**) Quantitative measurement of ONL thickness where *Prkab1*^+*/*+^ n = 11, average = 56.24 µm, SE = 1.37 and *Prkab1*^−/−^ n = 14, average = 52.57 µm, SE 1.12 *p*-value = .039. All error bars represent standard error of the mean, * indicates *p*-value < .05 result of student’s two-tailed t-test. Retinal layers abbreviated as above.

*Ap4e1*^−/−^ mice had ocular abnormalities in the retina, cornea, and lens. Corneal epithelia irregularity can be appreciated on gross exam of the eye (Fig. 4a). There is extensive multifocal retinal hypopigmentation on color fundus photography (Fig. 4b). There is abnormal lens morphology, decreased total retinal thickness, and reduced thickness of the photoreceptor outer segment layer, (Fig. 4c–f). On gross examination *Ap4e1*^−/−^ mice were noted as having abnormal kidney morphology, quantitative analysis of the kidneys revealed a decrease in overall weight. (Fig. 4g). *Dync1li1* homozygous KO mice are microphthalmic (Fig. 5b) and demonstrate a decreased total thickness and extensive retinal hypoplasia and atrophy with cellular disorganization across all layers of the retina histologically (Fig. 5a). Mice heterozygous for *Prkab1* (*Prkab*^+*/*−^) showed expression of LacZ under control of the *Prkab1* promoter in the photoreceptor outer segments (Fig. 6c) LacZ expression under control of the *Prkab1* promoter in male *Prkab1*^+/−^ mice reproductive tract is found in seminiferous tubule epithelium (Fig. 6a). Female reproductive tract has scattered lacZ expression present in the oviduct. (Fig. 6b). Mice homozygous null for *Prkab1* (*Prkab*^−/−^) have a decreased total retinal thickness (Fig. 7a,b) however do not have a reduction in size of the photoreceptor outer segment (Fig. 7c). *Prkab*^−/−^ do have a reduction in the thickness of the outer nuclear layer (Fig. 7d).

## Discussion

Ciliopathies are diseases of cilia, a cellular organelle in many eukaryotic cells. There are two distinct types of cilia; primary cilia and motile cilia, though all cilia have a microtubule-based axoneme structure covered by a plasma membrane that extends into the extracellular space. In humans, primary cilia enable cells to sense the environment. Motile cilia beat in a coordinated fashion to move cells or fluid external to cells. Primary cilia are found one per cell and are histologically distinct from motile cilia. The axoneme differs between primary and motile cilia. Primary cilia have a 9-microtubule doublet structure without central microtubules or a “9 + 0 structure”, while motile cilia have 9-microtubule doublets surrounding a two-microtubule “9 + 2 structure”. Motile cilia have dynein arms that connect the 9 microtubule doublets and allow the cilia to move through dynein motor activity^23^.

The phenotypic spectrum of ciliopathies reflect the function of both immotile and motile cilia^24^. The hallmark features of abnormal motile cilia are chronic respiratory conditions due to defective mucociliary clearance, infertility due to abnormal sperm motility or maturation^25^, and hydrocephalus as a result of defects in the ependymal cells that line the ventricles of the brain and are functionally responsible for normal production, resorption, and flow of cerebrospinal fluid^25^. Shared with both motile and immotile ciliopathies are *situs* abnormalities and congenital cardiac abnormalities. Motile and immobile cilia are expressed at the embryonic node during development and are responsible for determining left–right asymmetry by movement of morphogens^26^. Phenotypes unique to immotile or primary ciliopathies are broad and reflect the diverse environmental sensing and signaling function of the organelle. Renal and ocular abnormalities, specifically retinal abnormalities, are a common feature of ciliopathies. However, defects of the primary cilia can also include anosmia, hearing loss, obesity, skeletal anomalies, genital anomalies, various neurological disorders, craniofacial abnormalities, and liver disease^24^.

Ciliopathies affecting the eye typically result in retinal degeneration and include LCA, NPHP, SLS (a syndrome with main features of NPHP and LCA), JBTS, and BBS^8^. LCA is a retinal dystrophy that presents in infants. In humans, mutations in ciliary proteins RPGRIPL1 and CEP290 are linked to LCA and to several other ciliopathies^1^. NPHP commonly progresses to chronic kidney diseases within the first 30 years of life and is also associated with retinopathy resulting in SLS^27^. BBS has hallmark features of retinitis pigmentosa and kidney failure. Sixteen genes are thought to be associated with BBS and 8 of these make up an octameric complex of the ciliary basal body^28^. JBTS results in a cerebellar vermis hypoplasia and enlarged superior cerebellar peduncles along with mental retardation. Individuals with JBTS can also have retinal dystrophy and renal failure with nephronophthisis, which can result in normal or reduced kidney size^29,30^. Mutations of the same gene within the cilia can manifest with a varying constellation of clinical signs. For example, *NPHP1* is a gene that can be mutated in either NPHP^31^, SLS^32^, or JBTS^33^. It has been localized to the connecting cilium of the photoreceptor and its normal function is thought to be protein transport along the photoreceptor. Mice with mutations in *Nphp1* have abnormal intraflagellar transport along the cilia and have protein sorting defects along the inner and outer segments of the photoreceptor^34^.

Twenty-four of the 140 potential ciliopathy genes were identified as being direct cilia interactants (*Abi2, Ap4e1, Cdc42, Cdk4, Celsr1, Cops7a, Cyba, Dtnbp1, Dync1li1, Ehmt1, Folr1, Herc1, Klhl5, Mapt, Mib2, Myo7a, Prkab1, Ptp4a1, S1pr3, Sik3, Sugp1, Ube2a, Wsb2, and Xbp1*) based on STRING protein interaction analysis (Fig. 1b). Eight of the 140 potential ciliopathy genes had predicted secondary interaction with gold standard cilia proteins, *Aplnr, Casr, Gcgr, Grm6, Med1(or Mbd4), Mlh1, Rxfp2,* and *Wdr62.* (Fig. 1b,c). In total we identified a list of 32 candidate ciliopathy proteins, summarized in Table S1.

Retinal dysplasia, as seen in the *Abi2* KO mice, has been previously noted in the description of the ocular manifestations of Meckel syndrome^35,36^. *Abi2* KO mice have previously been shown to have defects in lens development, specifically abnormalities in secondary lens fiber migration^37,38^. Here we reproduce that *Abi2* may have a role in lens development. The IMPC *Abi2* homozygous KO mice had focally extensive swollen and disrupted lens fibers with abnormally retained nuclei (balloon cells) with capsular thickening and wrinkling in the cortical region of the lens (Fig. 2a). *Abi2* is thought to have a role in cytoskeleton remodeling at epithelial cell–cell junctions and dendritic spines^37^. The developing lens fiber cells have polarized primary cilia, but they are not thought to play an essential role in directing the formation of the lens^39^. Mutations in genes that are known ciliopathy genes such as *Bbs4* and *Bbs8* do not have an essential role in coordinating lens alignment^40^. *ABI2* or *Abi2* has not been shown to have any association with ciliopathies in humans nor links to retinal abnormalities characteristic of ciliopathies.

Human male and female infertility have been reported in ciliopathies. There are several cilia in the male reproductive system: sperm flagella, multiple motile cilia of the efferent ducts of the testis (rete testis) and efferent ductules of the epididymis and primary cilia of the testis, rete testis, epididymis, and prostate^41^. Furthermore, the multiple motile cilia of the efferent ductules of the epididymis have been shown to be important for clearing fluid and moving sperm by keeping them in suspension in fluid, failure of which leads to clogging and back flow of sperm into the testis^42^. The *Abi2* KO mice had small testes, which on histopathology showed testicular degeneration, marked, chronic, bilateral, multifocal vacuolation of the seminiferous tubular epithelium with hypocellularity, sparse spermatids, and very few spermatozoa tubular lumens. Apoptotic bodies and multinucleated giant cells were frequent. The epididymis showed marked chronic hypospermia. All segments of the epididymal ductules (caput, corpus, cauda) contained round bodies in the lumen with few spermatozoa. This suggests a failure in the maturation of sperm and subsequent immune mediated degradation of the sperm leading to testicular atrophy (Fig. 2c–e).

*Ap4e1* KO mice have been shown to specifically have retinal abnormalities of marked reordering of the outer plexiform layer. Other retinal abnormalities of *Ap4e1* KO mice include reduced responses in both the scotopic a-wave, which represents photoreceptor function, as well as the scotopic b-wave on ERG^38^. Here we recapitulate a different retinal phenotype for *Ap4e1* mutants consisting of decreased POS thickness (Fig. 4f) and decreased total retinal thickness (Fig. 4e), as well as abnormal lens morphology (Fig. 4c). Additionally, mutations in the human gene result in a pale optic disc. Other abnormalities include cerebral palsy, microcephaly, and intellectual disability^43^. This gene at the cellular level is a part of a heterotetrametric complex that mediates vesicle formation and sorting^44^. Though *Ap4e1* does not have an established role in cilia, the predicted protein interaction with OCRL1 is suggestive that *Ap4e1* may be involved in ciliary assembly as OCRL1 is known to be involved in primary ciliary assembly and mutations in *OCRL1* result in congenital cataracts, renal abnormalities, and learning disabilities, a ciliopathy syndrome called Lowe syndrome^45^.

*Wdr62* has evidence of being a novel second order ciliopathy gene through its interaction with an intermediary protein CEP63 (Fig. 1c). *Wdr62* KO mice had testicular degeneration (Fig. 3c) and epididymal hypospermia. All segments of epididymal ducts contained round bodies in the lumen with few spermatozoa (Fig. 3d). The retinal abnormalities, multifocal localized retinal dysplasia (Fig. 3a), along with the testes and epididymides changes are highly suggestive of defective ciliary function since both infertility and retinal abnormalities can be considered hallmarks of either motile or immotile ciliopathies^14^. A previously published *Wdr62* KO mouse strain established its role in centriole biogenesis^46^ and oocyte meiotic initiation^47^. WDR62 is a centriole associated protein and has been noted previously in the literature to interact with CEP63 as part of centriole duplication and when mutated can result in microcephaly^46^. The centrioles are responsible for forming the base of the axoneme, the “skeleton” or case that the cilia will grow out into^48,49^. Here we present evidence that *Wdr62* mutations can result in defective formation of the motile cilia as manifest by IMPC *Wdr62* homozygous KO mice with both aspermia and several malformations of the lens of the eye (Fig. 3a,c,d).

*Dync1li1* has a previously described KO mouse that reported a role for *Dync1li1* in both retinal and ciliary biology. These *Dync1li1* KO mice were reported to have increased photoreceptor degradation and a described role in stabilization of dynein during ciliogenesis. *Dync1li1* normally functions to promote Rab11-vesicle trafficking and efficient OS protein transport from Golgi to the basal body^50^. Here we confirm that *Dync1li1* KO mice have retinal defects and photoreceptor degradation demonstrated by atrophy at various levels of the retina (Fig. 5a). In addition, DYNC1LI1 may have a role in human cilia since it is predicted to interact with several known human cilia proteins (Fig. 1b).

*Prkab1* heterozygous KO mice have no previous described role in ciliary biology in vertebrates or humans (Table S1). A previously reported *Prkab1* KO mouse had splenomegaly, anemia, and erythrocyte morphologic abnormalities; a cluster of pathologies consistent with hemolytic anemia^51^. *Prkab1* is a part of the beta subunit of serine/threonine AMP-activated protein kinase, a heterotrimeric complex containing a catalytic alpha subunit paired with beta and gamma regulatory subunits. This protein complex is thought to be an essential regulator of cellular energy balance^52^. Expression was observed of the lacZ reporter under *Prkab1* native promoter throughout the male and female reproductive tracts in the testis and oviduct. (Fig. 6a–b) Further, expression was observed in the outer segments of the photoreceptor cells. (Fig. 6c). *Prkab1* homozygous KO mice show decreased total retina thickness (Fig. 7b), although the outer segment of the photoreceptor showed no difference in thickness compared to control. (Fig. 7c) *Prkab1* homozygous KO do show a reduction in the outer nuclear layer thickness. (Fig. 7d). Given that the outer nuclear layer contains the cell bodies of the photoreceptor cells^53^. Taken with the evidence of expression of the lacZ reporter under *Prkab1* native promote in the outer segments of the photoreceptor cells, this may suggest a role for *Prkab1* in development of the structure of the outer nuclear cell layer.

The results here provide both biologic and bioinformatic evidence of candidate ciliary disease genes that have mouse KO data to suggest a role in eye, kidney, and reproductive biology. The IMPC serves as a valuable resource of standardized KO mouse production and phenotyping to identify gene function. This approach can facilitate discovery of novel genes potentially associated with human disease, particularly those with multiple organ system involvement as each mouse undergoes comprehensive analysis of every major organ system. In combination with protein–protein interaction analysis, the IMPC data set provided us with a powerful tool for predicting gene function, multi-system disease processes, and biological processes based on predicted binding partners. However, this type of research has significant limitations. The pathologies present in a mouse KO may not be representative of human disease states due to species differences, which may negate disease relevance in humans. Also, the ability of the IMPC’s phenotyping pipeline to detect abnormalities is limited to the panel of tests that are used, and thus, phenotypes will be missed. Furthermore, interactions between proteins may be context dependent in a cell-type specific manner and may not be representative of the cellular function in every organ system. Mechanistic biochemical studies are required to confirm the ciliopathy candidates in this report and despite the overlapping ocular, renal, and reproductive abnormalities attributed to each knockout line in this study, direct validation of each candidate protein should be confirmed in primary cilia.

In summary, we present in vivo analytical findings for 32 candidate ciliopathy genes based on phenotypic changes identified in KO mice and predicted protein–protein interactions with known human cilia genes. One of the 32 has been shown previously to have function in human cilia, leaving 31 new candidate human ciliopathy gene candidates. Twenty-five of these are previously undescribed candidate vertebrate ciliary genes. Five of these genes predicted to have ciliary interaction (*Abi2, Wdr62, Ap4e1, Dync1li1, and Prkab1*) had abnormal morphological features detected by imaging that are consistent with the spectrum of phenotypes observed in ciliopathies. *Wdr62* and *Dyncli1* have previously described roles in vertebrate ciliary biology, while *Abi2, Ap4e1,* and *Prkab1* do not. *Abi2, Ap4e1,* and *Prkab1* therefore have strong evidence to be novel candidate ciliopathy genes. These candidate genes require validation in unsolved human cases with systemic features pertinent to ciliopathy. If relevant in human disease, these mouse KO may serve as valuable models for study of this group of ciliopathies.

## Materials and methods

### Knockout strain production

Detailed methods for mutant mouse production has been published previously^20^. For those lines generated from homologous recombination in ES cells, a *lacZ* reporter was integrated into the gene targeting vector to enable tissue-specific expression under the control of the endogenous promoter^54^. Phenotyping data was generated by the analysis of sex-balanced cohorts of homozygous (or heterozygous if embryo lethal) adult knockout female (n ≥ 7) and male (n ≥ 7) mice between 4 and 16 weeks of age and compared to contemporaneous data on age, sex (n = 2 male and n = 2 female), and genetic background (C57BL/6 N)-matched wildtype control mice. Strict ethical review and guidelines of accrediting authorities were followed by all International Mouse Phenotyping Consortium (IMPC) centers specific to their national and regional legislation and local institutional guidelines (Institutional Animal Care and Usage Committees, Regierung von Oberbayern, Com’Eth, Animal Welfare and Ethical Review Bodies, RIKEN Tsukuba Animal Experiments Committee, and Animal Care Committee). The IMPC Consortium collects data from international member institutes who collect phenotyping data guided by their own ethical review panels, licenses, and accrediting bodies that reflect the national and/or geo-political constructs in which they operate. We have captured this data via an ethical and funding survey from each contributing institute, this can be found at http://www.mousephenotype.org:8858/wp-content/uploads/2020/02/EthicalInfo2014.pdf. A comprehensive overview of the IMPC mouse phenotyping protocol consistent with ARRIVE guidelines^55^ can be found at https://www.mousephenotype.org/about-impc/animal-welfare/arrive-guidelines/ and is published previously^21^.

### Phenotyping

A comprehensive ophthalmic examination was performed on both eyes of each mouse at 15–16 weeks of age by experienced technical staff using ocular imaging equipment overseen by lead site scientists and/or expert ophthalmologists. Examiners were trained to identify and annotate background lesions (e.g., retinal dysplasia) commonly but inconsistently observed in the C57BL/6 N strain^56^. Fertility testing of both sexes was performed at 8–14 weeks, renal morphology was assessed during necropsy at 16 weeks, and clinical chemistry testing was done on a serum sample collected prior to termination.

As described in prior publications from the IMPC, phenotyping of mice was performed in a randomized fashion and technicians were blinded to the genotype (mutant or wildtype) of mice before and during examination^57^. Irises were pharmacologically dilated with 2.5% phenylephrine HCl (Akorn Inc., Lake Forest, IL, USA): 1% tropicamide (Bausch & Lomb Inc., Tampa, FL, USA) to facilitate eye examinations. A 0.1 mm slit beam at the highest intensity setting was used to evaluate the anterior segment including cornea, anterior chamber, and lens followed by assessment of the posterior segment including the vitreous chamber. The fundus was examined via indirect ophthalmoscopy using a 60 diopter double aspheric handheld lens (Volk Optical Inc, Mentor, OH, USA).

OCT imaging was performed (machine dependent on institution), after dilatation with both tropicamide 1% and phenylephrine 2.5%. Thickness of the total retina, inner nuclear layer, outer nuclear layer, were manually measured at a distance of ~ 0.2 mm from each side of the optic nerve using calipers in ImageJ software. Measurements from both sides of the optic nerve head and between left and right eyes were averaged for each animal.

Blood urea nitrogen and creatinine concentrations were analyzed on ~ 160–200 μl of plasma separated from whole blood collected by retro-orbital puncture in a gel tube containing lithium heparin after centrifugation for 10 min at 5000×*g* at 8 °C. Plasma samples were analyzed optimally on the day of collection in a clinical chemistry analyzer either Beckman Coulter AcT Diff, Siemens Advia 2120 or Hemavet Multispecies Hematology Analyzer HV950FS (Drew Scientific, CT, U.S.A.).

### Specimen collection

A complete necropsy was performed and all abnormal findings were recorded and annotated using the standardized IMPC Gross Pathology ontology^56^. Macroimages were captured of abnormal gross findings when possible. All fresh tissue samples collected at necropsy were immediately immersed in fixative (typically 10% neutral buffered formalin) and prepared for histopathological examination by a veterinary pathologist. Mild to moderate focal retinal dysplasia observed by histopathology were considered incidental background strain changes attributed to C57BL/6 N background and were excluded from genotype-associated phenotype calls. Parasagittal sections of eyes were sectioned at 5 µm, and stained with hematoxylin–eosin (H&E). Retinal layer measurements were taken of total retina, nerve fiber layer, retinal ganglion cell layer, inner plexiform layer, inner nuclear layer, outer plexiform layer, outer nuclear layer, photoreceptor outer segment, retinal pigmented epithelium, and choriod were manually measured at a distance of ~ 0.2 mm from each side of the optic nerve using calipers in NDP.view2. (U12388-01) (Hamamatsu, NJ, USA).

### Imaging

When possible at some centers with the requisite instrumentation, advanced imaging techniques were used to document suspected abnormalities for follow-up and evaluation. In these cases, mice were first anesthetized with an intraperitoneal injection of ketamine/midazolam (50–75/1–2 mg/kg) and their eyes dilated using topical tropicamide 1% and phenylephrine 2.5% drops and lubricated with artificial tears containing methylcellulose. Anterior segment and fundus images were acquired with a Micron III or IV retinal imaging microscope (Phoenix Research Laboratories, City, USA).

### Query of the IMPC database to identify potential ciliopathy genes

The IMPC website’s data release 11.0 was queried for five common abnormalities in human ciliopathy syndromes: abnormal ocular traits, abnormal renal morphology, abnormal renal function, abnormal reproductive morphology, and abnormal reproductive function. For “abnormal ocular traits” the term “abnormal ocular phenotypes” was used to search the database. Abnormal renal morphologies were identified by searching “abnormal kidney/renal phenotypes”. Abnormal renal function was identified by searching for “abnormal blood urea nitrogen (BUN)” or “abnormal blood creatinine (CRE)”, both commonly used lab values to assess kidney function in clinical practice^58^. Abnormal reproductive morphology was identified by query of the database for “abnormal reproductive morphology”. Abnormal reproductive function was identified by searching for “infertility” and its synonym “abnormal fertility/fecundity”.

To identify genes with the potential for being associated with a ciliopathy, all genes associated with abnormal renal morphology, abnormal renal function, abnormal reproductive morphology, and abnormal reproductive function were then assessed for any concomitant abnormal ocular traits. Each gene set for the aforementioned systemic trait abnormalities was compared for intersection, or overlapping genes, using Interactive Venn^59^ (Fig. 1a and Table S2). This list of genes with both ocular and renal or reproductive trait abnormalities are referred to as “potential ciliopathy genes”.

To provide quantitative data relating to retinal thickness, the current release of the IMPC 16.0 was queried on 6/1/2022 for the retina thickness data of *Ap4e1* and *Prkab1* heterozygote and homozygous KO mice.

### Bioinformatic analysis

To determine if the protein products of the “potential ciliopathy genes” interacted directly with any of the existing known ciliopathy genes, the mouse genes were transposed to their human orthologs. The gene list of 217 known human ciliopathy genes was previously generated, Boldt et al.^15^ reported a list of 217 proteins, where 124 of the 217 are Syscilia gold standard proteins^16^, 91 of the 217 are ciliopathy-associated proteins, and 80 of the 217 are proteins with predicted ciliary function, with genes overlapping between the three categories^60^. Protein interaction networks were built using the functional protein association network tool STRING-db^61^ version 1.5.1 built in Cytoscape 3.8 App, StringApp^62^. The “potential ciliopathy genes” and “known ciliopathy genes” were analyzed for protein–protein interactions using a confidence threshold of 0.9 (Fig. 1b). To further test if any of the “potential ciliopathy genes” had interactions with “known ciliopathy genes”, 35 interacting proteins predicted by StringDB which were not part of either of the “potential ciliopathy genes” list nor the 217 “known ciliopathy genes” list were included in the analysis (Fig. 1c).

### Literature review of candidate ciliopathy genes

Potential ciliopathy genes that had a predicted protein interaction with known ciliopathy genes either directly or through a secondary protein were considered to be “candidate” ciliopathy genes. All candidate ciliopathy genes were individually queried (www.pubmed.gov and www.google.com/scholar) with a search term (“kidney” “renal” “eye” “ocular” “retina” “reproductive” and “infertility”) to determine previous association between the respective trait abnormalities. The search was further modified to determine whether a KO mouse had been previously generated and analyzed. For candidate ciliopathy genes, gene names and the search terms “cilia” or “ciliopathy” were queried for previous implication in ciliary biology. In addition, MalaCards^63^ database was queried for each gene; those with publications supporting disease associations are summarized in Table S1.

### Statistical analysis

Protein interaction networks were built using the functional protein association network tool STRING-db^61^ version 1.5.1 built in Cytoscape 3.8 App, StringApp^62^. The “potential ciliopathy genes” and “known ciliopathy genes” were analyzed for protein–protein interactions using a confidence threshold of 0.9. Confidence threshold calculations done are a setting in the version 1.5.1 built in Cytoscape 3.8 App, StringApp.

Phenotyping data was generated by the analysis of sex-balanced cohorts of homozygous (or heterozygous if embryo lethal) adult knockout female (n ≥ 7) and male (n ≥ 7) mice between 4 and 16 weeks of age and compared to contemporaneous data on age, sex (n = 2 male and n = 2 female), and genetic background (C57BL/6 N)-matched wildtype control mice. Detailed methods for mutant mouse production has been published previously^20^.

### Ethics approval and consent to participate

Strict ethical review and guidelines of accrediting authorities were followed by all International Mouse Phenotyping Consortium (IMPC) centers specific to their national and regional legislation and local institutional guidelines (Institutional Animal Care and Usage Committees, Regierung von Oberbayern, Com’Eth, Animal Welfare and Ethical Review Bodies, RIKEN Tsukuba Animal Experiments Committee, and Animal Care Committee).

### Consent for publication

All authors have agreed to the publication of this article.

## Supplementary Information

Supplementary Information 1.

Supplementary Table S1.

Supplementary Table S2.

Supplementary Table S3.

## Data Availability

Materials Availability All mice generated are available from the IMPC. See impc.org for more details.

## Abbreviations

BBS: Bardet–Biedl Syndrome
BUN: Blood urea nitrogen
CRE: Blood creatinine
DCC: IMPC Data Coordination Center
HCl: Hydrogen Chloride
IMPC: International Mouse Phenotyping Consortium
JBTS: Joubert Syndrome
KO: Knockout
LCA: Leber Congenital Amaurosis
MGS: Meckel–Gruber syndrome
NPHP: Nephronophthisis
PCD: Primary cilia dyskinesia
PKD: Polycystic kidney disease
SLS: Senior-Loken Syndrome
RNFL/GCL: Retinal nerve fiber layer/ganglion cell layer
IPL: Inner plexiform layer
INL: Inner nuclear layer
OPL: Outer plexiform layer
ONL: Outer nuclear layer
POS: Photoreceptor outer segments
RPE: Retinal pigmented epithelium
C: Choroid

## Code availability

All raw data is available at impc.org, no original code was generated during the research.
