# Supplementary material for: Analysis of genome-wide knockout mouse database identifies candidate ciliopathy genes
Source: Sci Rep. 2022 Dec 1;12:20791. doi: 10.1038/s41598-022-19710-7 (PMC9715561; doi:10.1038/s41598-022-19710-7)
Supplement: Supplementary file 1 — Supplementary Information 1. [file 41598_2022_19710_MOESM1_ESM.pdf]

INTERNATIONAL MOUSE PHENOTYPING CONSORTIUM MEMBERS

David Adams[14], Arthur L. Beaudet[12], Fatima Bosch[27], Bob Braun[15], Steve Brown[11], Hsian-Jean Genie Chin[31], Mary Dickinson[12] , Michael Dobbie[28], Paul Flicek[23], Sanjeev Galande[29], Xiang Gao[21], Anne Grobler[30], Jason D. Heaney[12], Yann Herault[16,17,18,19,20], Martin Hrabe[13], Natasha Karp[14], Ann-Marie Mallon[11], Fabio Mammano[26], Terrence Meehan[23], Yuichi Obata[22], Helen Parkinson[23], Chuan Qin[32], Radislav Sedlacek[33], Toshihiko Shiroishi[22], JK Seong[25] Damian Smedley[24], Glauco Tocchini-Valentini[26], Sara Wells[11], Ying Xu[34].

[11] Medical Research Council Harwell Institute (Mammalian Genetics Unit and Mary Lyon Centre), Harwell, Oxfordshire OX11 0RD, UK.
[12] Department of Molecular and Human Genetics, Baylor College of Medicine, Houston, TX 77030, USA.
[13] German Mouse Clinic, Institute of Experimental Genetics, Helmholtz Zentrum München, German Research Center for Environmental Health, Ingolstädter Landstraße 1, 85764 Neuherberg, Germany.
[14] The Wellcome Trust Sanger Institute, Wellcome Genome Campus, Hinxton, Cambridge CB10 1SA, UK.
[15] The Jackson Laboratory, Bar Harbor, ME 04609, USA.
[16] Institut de Génétique et de Biologie Moléculaire et Cellulaire, Université de Strasbourg, 1 rue Laurent Fries, 67404 Illkirch, France.
[17] Centre National de la Recherche Scientifique, UMR7104 Illkirch, France.
[18] Institut National de la Santé et de la Recherche Médicale, U1258 Illkirch, France.
[19] Université de Strasbourg, 1 rue Laurent Fries, 67404 Illkirch, France.
[20] CELPHEDIA, PHENOMIN, Institut Clinique de la Souris (ICS), CNRS, INSERM, Université of Strasbourg, 1 rue Laurent Fries, 67404 Illkirch-Graffenstaden, France.
[21]SKL of Pharmaceutical Biotechnology and Model Animal Research Center, Collaborative Innovation Center for Genetics and Development, Nanjing Biomedical Research Institute, Nanjing University, Nanjing 210061, China.
[22] RIKEN BioResource Center, Tsukuba, Ibaraki 305-0074, Japan.
[23] European Molecular Biology Laboratory, European Bioinformatics Institute, Wellcome Genome Campus, Hinxton, Cambridge CB10 1 SD, UK.
[24] Clinical Pharmacology, Charterhouse Square, Barts and the London School of Medicine and Dentistry, Queen Mary University of London, London EC1M 6BQ, UK.
[25] Korea Mouse Phenotyping Consortium (KMPC) and BK21 Program for Veterinary Science, Research Institute for Veterinary Science, College of Veterinary Medicine, Seoul National University, 599 Gwanangno, Gwanak-gu, Seoul 08826, South Korea.
[26] Monterotondo Mouse Clinic, Italian National Research Council (CNR), Institute of Cell Biology and Neurobiology, Adriano Buzzati-Traverso Campus, Via Ramarini, I-00015 Monterotondo Scalo, Italy.
[27]Universitat Autonoma de Barcelona, Barcelona, Spain.
[28] Phenomics Australia, The Australian National University, 131 Garran Rd, Acton ACT 2601, Australia.
[29]Indian Institutes of Science and Education Research, Dr Homi Bhabha Rd, Ward No. 8, NCL Colony, Pashan, Pune, Maharashtra 411008, India
[30]PCDDP North-West University North-West University Potchefstroom Campus 11 Hoffman Street Potchefstroom 2531
[31]National Laboratory Animal Center, National Applied Research Laboratories 3F., No. 106, Sec. 2, Heping E. Rd., Da'an Dist., Taipei City 106214, Taiwan (R.O.C.)
[32]Institute of Laboratory Animal Sciences, Chinese Academy of Medical Science 5 Panjiayuan Nanli, Chaoyang District, Beijing, 100021, China
[33] Czech centre for phenogenomics, IMG BIOCEV building SO.02 Prumyslova 595 252 50 Vestec, Czech Republic, Europe.

[34]CAM-SU Genomic Research Center, Soochow University, Organization Planning of No. 1 Shizi Street, Suzhou, China.

IMPC Full List of Members

Cheryl Ackert-Bicknell, David J Adams, Douglas Adams, Anne-Tounsia Adoum, Juan A Aguilar-Pimentel, Dalila Ali-Hadji, Oana V Amarie, Philippe André, Aurelie Auburtin, Chaouki Bam'Hamed, Johannes Beckers, Joachim Beig, Alexandr Bezginov, Marie-Christine Birling, Katharina Boroviak, Joanna Bottomley, Lynette Bower, Mohammed Bubshait, Antje Bürger, Dirk H Busch, Natalie C Butterfield, Jorge Cabezas, Pilar Cacheiro, Julia Calzada-Wack, Emma L Cambridge, Marie-France Champy, Tracy Carroll, Heather Cater, Philippe Charles, Elissa J Chesler, Yi-Li Cho, Valentina Cipriani, Greg Clark, Shannon Clarke, Nicola Cockle, Gemma Codner, Amie Creighton, Maribelle Cruz, Katharine F Curry, Abigail D'Souza, Ozge Danisment, Daniel Delbarre, Hannah F Dewhurst, Brendan Doe, Alex Dorr, Florian Giesert, Graham Duddy, Kyle Duffin, Amal El Amri, Hillary Elrick, Patricia Feugas, Martin Fray, Anthony Frost, Helmut Fuchs, Valerie Gailus-Durner, Karen K Gampe, Milan Ganguly, David Gannon, Lillian Garrett, Marina Gertsenstein, Diane Gleeson, Leslie Goodwin, Jochen Graw, Kristin Grimsrud, Ruolin Guo, Hamed Haselimashhadi, Jason D Heaney, Liane Hobson, Andreas Hörlein, Deborah Hogg, Sabine M Hölter, Seung-Hyun Hong, Neil Horner, Ziyue Huang, Jane Hunter, Joanna Joeng, Coleen Kane, Lois Kelsey, Janet Kenyon, Ruairidh King, Piia Keskivali-Bond, Andrea Kirton, Tanja Klein-Rodewald, Thomas Klopstock, Davide Komla-Ebri, Tomasz Konopka, Ralf Kühn, Fiona Kussy, David Lafont, Qing Lan, Denise G Lanza, Valerie Laurin, Elise Le Marchand, Sophie Leblanc, Victoria D Leitch, Christoph Lengger, Lauri Lintott, John G Logan, Isabel Lorenzo, Suzanne MacMaster, Ann-Marie Mallon, Naila S Mannan, Susan Marschall, Matthew Mckay, Robbie SB McLaren-Jones, Jeremy Mason, Terrence F Meehan, David Miller, Michayla Moore, Lily Morikawa, Violeta Munoz-Fuentes, Stephen A Murray, Dong Nguyen-Bresinsky, Celeste Owen, Oskar Oritz, Panos Pandis, Helen Parkinson, Alexandru Parlog, Amit Patel, Guillaume Pavlovic, Patricia Penton, Monica Pereira, Kevin Peterson, Vivek Philip, Andrea S Pollard, Jan Prochazka, Dawei Qu, Sean Rangarajan,

Birgit Rathkolb, Mike Relac, Kyle Roberton, Willson Roper, Stéphane Rousseau, David W Rowe, Jan Rozman, Jennifer Ryan, Edward J Ryder, Luis Santos, Adrián Sanz-Moreno, Joel Schick, Zachary Seavey, John R Seavitt, Claudia Seisenberger, Mohammed Selloum, Xueyuan Shang, Dong-Guk Shin, Michelle Simon, Gillian Sleep, Damian Smedley, Tania Sorg, Penny C Sparkes, Nadine Spielmann, Ralph Steinkamp, Michelle Stewart, Claudia Stoeger, Ewan Straiton, Karen L Svenson, Holly Swash, Lydia Teboul, Sandra Tondat, Irina Treise, Catherine Tudor, Rachel Urban, Valerie E Vancollie, Laurent Vasseur, Igor Vukobradovic, Hannah Wardle-Jones, Jonathan Warren, Marie Wattenhofer-Donze, Sara E Wells, Henrik Westerberg, Jacqueline K White, Jean-Paul Wiegand, Amelia Willett, Catherine Witmeyer, Eckhard Wolf, Joshua Wood, Wolfgang Wurst, Catherine Xu, Yingchun Zhu, and Annemarie Zimprich.
